# Development of a Novel Lateral Flow Biosensor Combined With Aptamer-Based Isolation: Application for Rapid Detection of Grouper Nervous Necrosis Virus

**DOI:** 10.3389/fmicb.2020.00886

**Published:** 2020-05-19

**Authors:** Jiaxin Liu, Qiwei Qin, Xinyue Zhang, Chen Li, Yepin Yu, Xiaohong Huang, Omar Mukama, Lingwen Zeng, Shaowen Wang

**Affiliations:** ^1^Joint Laboratory of Guangdong Province and Hong Kong Region on Marine Bioresource Conservation and Exploitation, College of Marine Sciences, South China Agricultural University, Guangzhou, China; ^2^Guangdong Laboratory for Lingnan Modern Agriculture, College of Marine Sciences, South China Agricultural University, Guangzhou, China; ^3^Laboratory for Marine Biology and Biotechnology, Qingdao National Laboratory for Marine Science and Technology, Qingdao, China; ^4^Key Laboratory of Regenerative Biology, South China Institute for Stem Cell Biology and Regenerative Medicine, Guangzhou Institutes of Biomedicine and Health, Chinese Academy of Sciences, Guangzhou, China; ^5^School of Food Science and Engineering, Foshan University, Foshan, China

**Keywords:** marine fish, RGNNV, detection, aptamer, lateral flow biosensor

## Abstract

Nervous necrosis virus (NNV) has infected more than 50 fish species worldwide, and has caused serious economic losses in the aquaculture industries. However, there is no effective antiviral therapy. The development of a rapid and accurate point-of-care diagnostic method for the prevention and control of NNV infection is urgently required. Commonly used methods for NNV detection include the cell culture-based assay, antibody-based assay and polymerase chain reaction (PCR)-based assay. However, these methods have disadvantages as they are time-consuming and complex. In the present study, we developed a simple and sensitive aptamer-based lateral flow biosensor (LFB) method for the rapid detection of red-spotted grouper nervous necrosis virus (RGNNV). An aptamer is a single-stranded nucleotide, which can specifically bind to the target and has many advantages. Based on a previously selected aptamer, which specifically bound to the coat protein of RGNNV (RGNNV-CP), two modified aptamers were used in this study. One aptamer was used for magnetic bead enrichment and the other was used for isothermal strand displacement amplification (SDA). After amplification, the product was further tested by the LFB, and the detection results were observed by the naked eye within 5 min with high specificity and sensitivity. The LFB method could detect RGNNV-CP protein as low as 5 ng/mL or 5 × 10^3^ RGNNV-infected GB (grouper brain) cells. Overall, it is the first application of a LFB combined with aptamer in the rapid diagnosis of virus from aquatic animals, which provides a new option for virus detection in aquaculture.

## Introduction

Nervous necrosis virus (NNV), belongs to betanodavirus of the family *Nodaviridae*, is one of the smallest known animal viruses, about 25–30 nm in size (Guo et al., 2004). NNV mainly destroys the central nervous system of lower vertebrates, resulting in conspicuous vacuolation observed in the retina and brain, so viral nervous necrosis (VNN) is also called viral encephalopathy and retinopathy (VER) (Chi et al., 1997; Doan et al., 2016). The mortality of NNV infected larvae and juvenile fish can be 100% within 1 week. Importantly, vertical propagation of NNV between parents and their offspring was found in NNV infection (Johans et al., 2002). Because of its small size and rapid pathogenicity, it is a serious threat to aquaculture worldwide. However, at present, there is no effective method for curing NNV infection. There are four genotypes of NNVs, including red-spotted grouper nervous necrosis virus (RGNNV), striped jack nervous necrosis virus (SJNNV), barfin flounder nervous necrosis virus (BFNNV) and tiger puffer nervous necrosis virus (TPNNV). Among these viruses, RGNNV is the most serious, and can infect most marine fishes cultured in Asia, Europe, Australia and North America, leading to huge economic losses (Nishizawa et al., 1997; Harikrishnan et al., 2011; Shetty et al., 2012). Thus, it is essential to establish diagnostic methods for early detection and prevention of NNV infection.

In recent years, many methods of NNV detection have been developed, including the cell culture-based assay, antibody-based assay and polymerase chain reaction (PCR)-based assay. The cell culture-based assay is the most basic method for NNV diagnosis, but it is time-consuming and low sensitivity, and must require professional laboratory and sensitive cell lines (Frerichs et al., 1996; Hick et al., 2011; Toubanaki and Karagouni, 2017). As the standard method, PCR is much more popular (Mekata et al., 2014). Traditional PCR has grown rapidly and several different forms have been developed, such as reverse transcription PCR (RT-PCR) (Dalla Valle et al., 2000; Mu et al., 2013), quantitative real-time PCR (qRT-PCR) (Dalla Valle et al., 2005; Cherif et al., 2011), and loop-mediated isothermal amplification (LAMP) (Xu et al., 2010; Mekata et al., 2014). Although PCR is widely used, it also has limitations as it requires complex procedures and specialized equipment, and it cannot be used on the spot. Other methods, such as electron microscopic observation of infected tissues (Nopadon et al., 2009), enzyme-linked immunosorbent assay (ELISA) (Arimoto et al., 1992; Shieh and Chi, 2005; Nuñez-Ortiz et al., 2016), and immuno-fluorescence antibody test (IFAT) (Nguyen et al., 1996; Mladineo, 2003), require well-trained personnel and expensive instruments. They are also laboratory-based and do not meet the requirements for real-time detection. Consequently, effective and convenient methods for sensitive detection still remain a challenge.

A lateral flow biosensor (LFB), also known as a dipstick test strip, is currently used for point-of-care detection. Different labels are used in a LFB, and the most widely used label is gold nanoparticles as the color can be seen by the naked eye (Posthuma-Trumpie et al., 2009; Pyo and Yoo, 2012; Ren et al., 2014). LFBs have been established for the detection of various targets, such as cells, heavy metals, microRNA, viruses and bacteria (Liu et al., 2009; Chai et al., 2010; Wu et al., 2013; Mdluli et al., 2014; Gao et al., 2016; Huang et al., 2016; Jiao et al., 2019; Li et al., 2020). LFBs have developed rapidly in recent years due to their low-cost, portability and specificity. An antibody is the traditional component of a LFB (Panferov et al., 2017; Shyam et al., 2020), but antibody preparation is complicated and there are quality differences in each batch. In recent years, the emergence of aptamers has provided a new and ideal choice for virus detection.

Aptamers, mainly selected using the process called systematic evolution of ligands exponential enrichment (SELEX), are single-stranded DNA or RNA (DNA aptamers have higher stability than RNA aptamers) (Tuerk and Gold, 1990; Rotherham et al., 2012). Based on their three-dimensional folding structure, they are similar to antibodies and show high specific affinity to bind to a wide range of targets including organic molecules, proteins, cells and metal ions (Tuerk and Gold, 1990; Ciesiolka et al., 1995; Liu et al., 2009). In addition, aptamers have good chemical stability, and can be synthesized in high purification with convenient modification and at low cost, so aptamers are now increasingly used in scientific research and diagnosis. Recently, new LFB combined with aptamers has been developed for pathogen detection, such as salmonella (Fang et al., 2014), *Escherichia coli* O157:H7 (Wu et al., 2015), hepatitis C virus (Wang et al., 2013), and influenza virus (Le et al., 2017). However, LFB combining with aptamer is still rarely applied in virus detection presently. Especially, there is no report of LFB combined with aptamer in detection of aquatic animal virus so far.

In our previous work, aptamers targeting the coat protein of RGNNV (RGNNV-CP) were selected (Zhou et al., 2016). Herein, we combined LFB and aptamers to establish a convenient and simple biosensor for RGNNV detection. In this study, two aptamers were incubated with the sample and then enriched with magnetic beads. If RGNNV was present, the strand displacement amplification (SDA) reaction was carried out. Ultimately, the amplification products of the SDA reaction were captured by the LFB to form visible bands. Using this method, we could detect RGNNV-CP as low as 5 ng/mL or 5 × 10^3^ RGNNV-infected GB (grouper brain) cells. To the best of our knowledge, this is the first report of a LFB combined with aptamer developed for the rapid diagnosis of NNV. This system also has low costs and simple operation, and showed good potential application in field tests.

## Materials and Methods

### Reagents and Chemicals

Oligonucleotides were synthesized and purified with high performance liquid chromatography by Invitrogen Biotechnology Co., Ltd. (Shanghai, China), and the sequences used in this study are listed in Table 1. HAuCl_4_ and bovine serum albumin (BSA) were purchased from Sigma Aldrich (United States). The polymerase Klenow fragment exo- and Nt.*Bbv*CI were purchased from New England Biolabs (United States). Deoxynucleoside triphosphates (dNTPs) were purchased from Takara (Japan). Streptavidin (SA)-modified magnetic beads (Dynabeads^TM^ MyOne^TM^ Streptavidin C1) were purchased from Invitrogen (United States). A portable strip reader (TSR-100) was purchased from Hangzhou Allsheng Instruments Co., Ltd. (Hangzhou, China). Nitrocellulose membranes (M 135) were purchased from Millipore (United States). Glass fiber (8 #) and absorbent pads (5 #) were purchased from Allway Biotech Co., Ltd. (Guangzhou, China). The SV Total RNA Isolation System was purchased from Promega (United States) and the ReverTra Ace qPCR RT Kit was purchased from Toyobo (Japan). All other chemicals used in this study were purchased from standard commercial sources and were analytical reagent grade. Buffer solutions were prepared with ultrapure water (18.2 MΩ/cm, Millipore, United States) in our laboratory.

**TABLE 1:** Oligonucleotide sequences used in this study.

**Oligonucleotide**	**Sequence (5′–3′)**
A-aptamer	TTCTTTTATTAGTTGATTTTTTTGATTTTGGCAGCTACTGC
	TTTGGGGGT^1^***GCTGAGG***^2^CGAAGGACGCAGATGAAGTCTC
A-aptamer-ELASA	TTCTTTTATTAGTTGATTTTTTTGATTTTGGCAGCTACTGC
	TTTGGGGGTGCTGAGGCGAAGGACGCAGATGAAGTC TC-^3^Bio
C-aptamer	TGTGTGTCTATTGTCTGTGGTTCATGTCAAGCTTATTTTCC
	ACACACGGT-^3^Bio
AuNP probe 1	^4^SH-AGCTACTGCTTTGGGGGT
C-line probe 2	ACCCCCAAAGCAGTAGCT
T-line probe 3	TTCTTTTATTAGTTGATTT
SDA-primer	GAGACTTCATCTGCGTCCTTCG
RGNNV-CP-RT-F	CAACTGACAACGATCACACCTTC
RGNNV-CP-RT-R	CAATCGAACACTCCAGCGACA
Actin-RT-F	TACGAGCTGCCTGACGGACA
Actin-RT-R	GGCTGTGATCTCCTTCTGCA

*^1^Bold italic characters indicate the recognition site sequence of Nt.*Bbv*CI. ^2^Solid underlines indicate the additional primer binding sequence. ^3^Bio indicates a 3′ biotin modification. ^4^SH indicates a 5′ thiol modification.*

### Cells, Virus, Proteins, and Aptamers

Grouper brain (GB) and grouper spleen (GS) cells were cultured at 28°C in Leibovitz’s L15 medium, and fathead minnow (FHM) cells were cultured in M199 medium, both containing 10% fetal bovine serum (FBS) (Huang et al., 2009, 2011). Red-spotted grouper nervous necrosis virus, Singapore grouper iridovirus (SGIV), and Largemouth Bass virus (LMBV) were prepared and stored in our laboratory at −80°C until use (Qin et al., 2006; Huang et al., 2011, 2014). The CP of RGNNV was prepared as described previously (Zhou et al., 2016). In brief, we amplified and cloned RGNNV-CP gene into the expression vector pMAL^TM^-c2X (New England Biolabs, Ipswich, MA, United States) and the recombinant MBP (Maltose binding protein)-CP fusion protein was expressed and purified by its MBP tag. To obtain test samples, cells were separately infected with RGNNV, SGIV and LMBV at the indicated time post-infection. At 36 h post-infection, the cytopathic effect (CPE) was observed with light microscopy. The infected cells were lysed in PierceIP^TM^ Lysis buffer (Thermo Fisher Scientific, United States), collected and washed three times with phosphate buffered saline (PBS, 137 mM NaCl, 2.7 mM KCl, 10 mM Na_2_HPO_4_, 1.8 mM KH_2_PO_4_, pH 7.4), and the lysates were used for further study. The uninfected cells were used as the control.

Two aptamers (A10 and B11) against RGNNV-CP were used in this study (Zhou et al., 2016). Aptamer binding affinities (Kd) to the RGNNV-CP were 4 nM for A10 and 6 nM for B11. The oligonucleotide sequences used are listed in Table 1. Biotin-labeled A10 was used as the capture aptamer (C-aptamer) and was captured by SA coated magnetic beads for recognizing and enriching targets. As the amplification-aptamer (A-aptamer), B11 was added two special sequence for SDA reaction. One added sequence in B11 acts as the target site for Nt.*Bbv*CI enzyme, and the other sequence was the binding site of the primer used in SDA reaction.

### Assembly of the Enzyme-Linked Apt-Sorbent Assay (ELASA)

We performed the ELASA with some modifications to identify the ability of the A-aptamer targeting the RGNNV-CP. The ELASA plates (42592 #, Corning Incorporated, United States) were pre-coated with RGNNV-CP (2 μg/mL) in advance, and maintained at 4°C in PBS overnight. Because the RGNNV-CP were purified by its MBP tag, then MBP was used as a control. 100 μL of 3% BSA was added to the plate wells for a block and then incubated at 4°C for 30 min, followed by washing three times with PBS. The biotin-labeled A-aptamer was denatured at 92°C for 8 min, and subsequently placed on ice for another 5 min. 500 nM biotin-labeled A-aptamer in 100 μL PBS was added to each well for 30 min at 4°C. After thorough washing with 200 μL of PBS three times, 100 μL of horse-radish peroxidase (HRP)-SA (Pierce HRP-Strep, Thermo Fisher Scientific; 1:10,000 dilution) was added and reacted for 30 min without light. Each well was further washed three times, and the bound HRP conjugates were detected according to the instructions of the Pierce TMB Substrate Kit (Thermo Fisher Scientific), and then the OD450 values were measured.

### Synthesis of Gold Nanoparticles (AuNPs)

The AuNPs with an average diameter of 15 nm were synthesized using a previously reported method with slight modifications (Fang et al., 2014). Briefly, 200 mL HAuCl_4_ solution (0.01%) was gently stirred and heated to boiling in a 500-mL round bottom flask, 8 mL of 1% trisodium citrate was quickly added to this solution, and the color turned deep blue in the first 20 s and then turned wine red within 60 s. The solution was boiled for an additional 5 min and then cooled to room temperature with gentle stirring for another 15 min. The resulting AuNPs were collected and stored at 4°C for further use.

### Preparation of AuNPs–DNA Conjugates

To prepare AuNPs–DNA conjugates, AuNPs were firstly concentrated 10 times by centrifugation (12000 rpm, 20 min), and then 10 μM of thiolated DNA (AuNP probe 1) was added to 500 μL of the 10-fold concentrated AuNPs solution (0.1%). The mixture was shaken gently overnight at 4°C. Following the addition of 10% BSA for 4 h, the DNA coated AuNPs solution was subjected to “aging” by the addition of 1% sodium dodecyl sulfate (SDS) and 1.5 M NaCl to a final concentration of 0.01% and 150 mM, respectively. The solution was kept at 4°C for another 24 h. Particles were centrifuged for 20 min at 12000 rpm and rinsed three times with rinsing buffer (20 mM Na_3_PO_4_, 5% BSA, 0.25% Tween and 10% sucrose) to remove excess unbound DNA. The supernatant was discarded and the red pellet was re-suspended in 150 μL of rinsing buffer, and the AuNP–DNA conjugate solution was then stored at 4°C until use (Mao et al., 2009; Wu et al., 2015).

### Assembly of the Lateral Flow Biosensor

**FIGURE 1:**
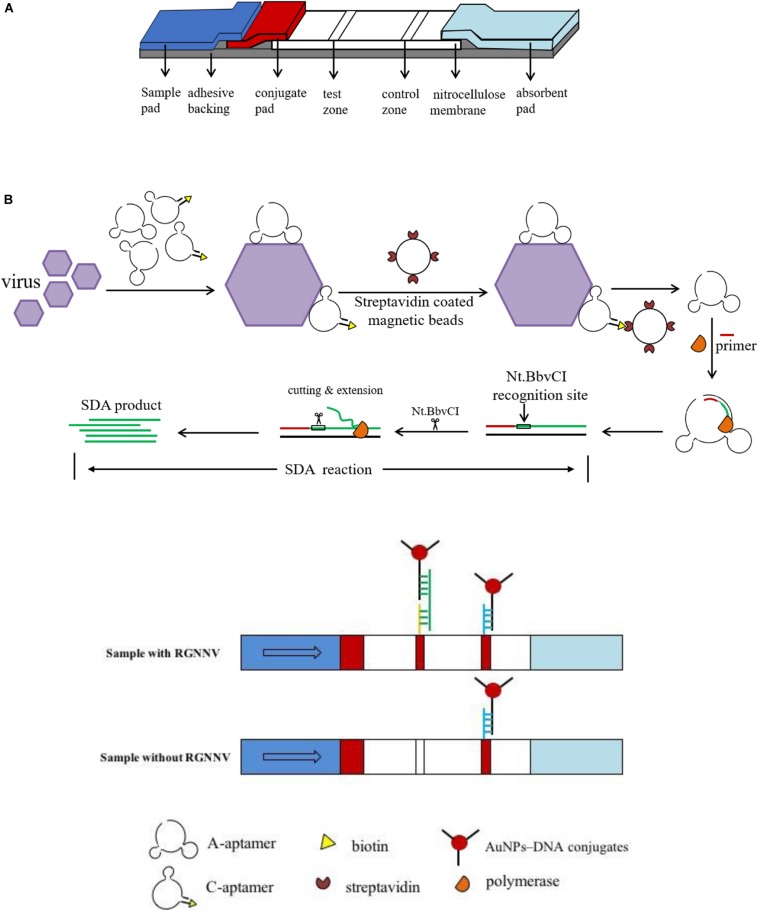
Schematic illustration of the lateral flow biosensor. **(A)** Assembly of lateral flow biosensor. A sample pad, conjugate pad, nitrocellulose membrane and absorbent pad were assembled on a plastic adhesive backing. **(B)** The procedure for RGNNV detection. Two aptamers were incubated with samples, then SA coated magnetic beads were added to the mixture to form A-aptamer-RGNNV-C-aptamer-SA coated magnetic beads complex. Collected by a magnet separator, the complex was transferred to a new tube to conduct the SDA reaction. After SDA, the product was loaded onto the sample pad of the biosensor.

A schematic of the fabrication of the LFB is shown in Figure 1A. A sample pad, conjugate pad, nitrocellulose membrane and absorbent pad were assembled on a plastic adhesive backing (60 mm wide × 30 cm long) with an overlap of 2 mm wide. The 4 mm wide strips were then cut using a paper cutter. The sample pad was soaked in sample pad buffer (0.5% Triton, 2% sucrose, 1% BSA, 50 mM boric acid, pH 8.0), kept at room temperature overnight for drying, and stored at 4°C before assembly. The C-line probe 2 (100 μM, 30 μL) and T-line probe 3 (100 μM, 30 μL) were dispensed on the nitrocellulose membrane simultaneously using a three-dimensional Scribing dispenser (Shanghai Kinbio, China) to form the control line and test line.

### Procedure for RGNNV Detection

The detection procedure is shown in Figure 1B, and the detailed process was briefly illustrated as follows. The C-aptamer and A-aptamer were incubated with RGNNV in 100 μL PBS for 1 min. The final concentration of each aptamer was 200 nM. Then 2 μL pre-washed SA coated magnetic beads were added to the above mixture with intermittent shaking for 5 min. The A-aptamer-RGNNV-C-aptamer-SA coated magnetic beads complex was collected by a magnet separator. After washing three times with PBST (PBS supplied with 0.1% Tween-20), the complex was resuspended by ultrapure water and transferred to a new tube to conduct the SDA reaction. Thereinto, the A-aptamer of the complex was used as the template. As shown in Table 2, the SDA reaction is mainly comprised of primer, Nt.*Bbv*CI enzyme, Klenow Fragment exo-DNA polymerase, incubated at 37°C for 30 min. The Nt.*Bbv*CI recognition site in the sequence of the modified A-aptamer was designed for SDA amplification. The amplification was initiated by the SDA-primer hybridizing with the additional primer binding sequence in the modified A-aptamer, followed by the DNA polymerase extension. The newly synthesized complementary strand was displaced by Nt.*Bbv*CI at the recognition site for another round of extension. Through the repeated process, a large amount of amplified single-stranded DNA (ssDNA) was produced, which was loaded onto the sample pad of the biosensor. Following saline-sodium citrate (0.6 M NaCl, 0.06 M sodium citrate, pH 7.0) rinsing, the test line and control line of the biosensor were easily observed within 5 min.

**TABLE 2:** Reaction system of SDA used in this study.

**Materials**	**Volume/μL**
SDA primer (10 μM)	2
dNTPs (25 mM)	2
Nt. *Bbv*CI (10000 U/mL)	0.4
Polymerase Klenow fragment exo-(5000 U/mL)	0.6
Polymerase Klenow fragment exo-reaction buffer-2 (10X)	2
BSA (10%)	1
Ultrapure water	12

### Sample Preparation for LFB and PCR Detection

Twenty groupers (8–10 cm in length) with suspected RGNNV infection were collected from farms in Hainan Province, China. Groupers were decapitated, and the brain tissue from each grouper was obtained and divided into two parts, one for LFB detection, and the other for RNA extraction.

The brain tissue was removed into 200 μL of protein lysis buffer (87787 #, Pierce^TM^ IP Lysis Buffer, Thermo Fisher), minced into small pieces (approximately 1 mm^3^) using surgical scissors, and then lysed at 4°C for 10 min. After centrifugation for 5 min at 4000 rpm, the supernatant was collected for LFB detection.

Total RNA from brain tissue was extracted using the SV Total RNA Isolation System (Promega, United States) and reverse transcription was carried out with the ReverTra Ace qPCR RT Kit (Toyobo, Japan) according to the manufacturer’s instructions. Primers used for RT-PCR detection were listed in the Table 1 according to the previous report (Huang et al., 2015).

## Results

### The Modified A-Aptamer Could Still Target RGNNV-CP

**FIGURE 2:**
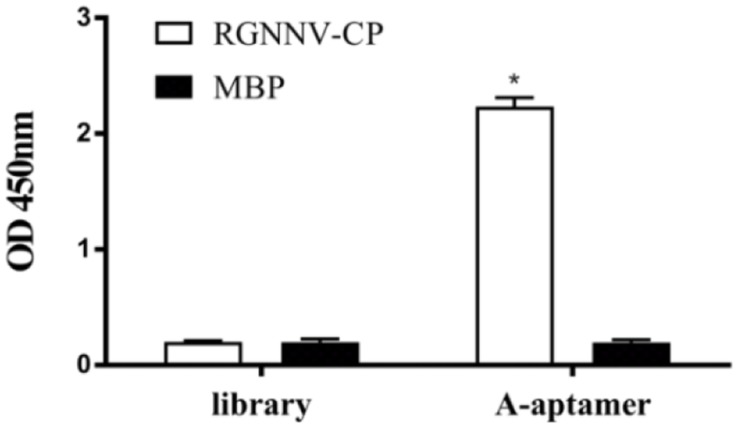
ELASA results showed the specific binding of modified A-aptamer to the RGNNV-CP. MBP and a random ssDNA library served as controls. OD_450_ values increased significantly of modified A-aptamer and RGNNV-CP, there was no obvious increase in the OD_450_ values of controls. Bars represented the means ± SD (*n* = 3). *p < 0.05.

As we modified the A-aptamer with a recognition site for Nt.*Bbv*CI and an additional primer binding sequence for SDA amplification, secondary structures of aptamers (B11 and modified-B11) were predicted using the MFold program (Zuker, 2003). Supplementary Figure 1 demonstrated that the aptamer with insertion of the additional sequences still retained the two stem-loop structures at 4°C with the ionic conditions of 157 mM Na^+^. Then we used ELASA to confirm the binding ability of the modified A-aptamer and RGNNV-CP. MBP and a random ssDNA library served as controls. As shown in Figure 2, the OD_450_ value increased obviously in modified A-aptamer and RGNNV-CP group compared to control groups. The results demonstrated that the modified A-aptamer could still specifically bind to the RGNNV-CP.

### Characterization of Synthesized AuNPs

The quality of synthesized AuNPs was measured by visual detection, UV spectrophotometry and transmission electron microscopy (TEM, Talos F200S, Thermo Fisher Scientific). From Supplementary Figure 2A, it can be seen that the AuNPs had a transparent bright red color with no precipitation on the bottom. Supplementary Figure 2B shows that an absorption peak appeared at 520 nm using UV spectrophotometry. The narrow width of the main peak illustrated that the particles in the AuNPs solution were well-distributed. The shapes and sizes of the AuNPs produced were observed by TEM and analyzed by Image J software. As shown in Supplementary Figure 2C, AuNPs were approximately 15 nm (15.3 ± 0.7 nm) in diameter and spherical in shape. The synthesized AuNPs were of good quality and were used for further study.

### Validation of the Biosensor for RGNNV-CP Detection

**FIGURE 3:**
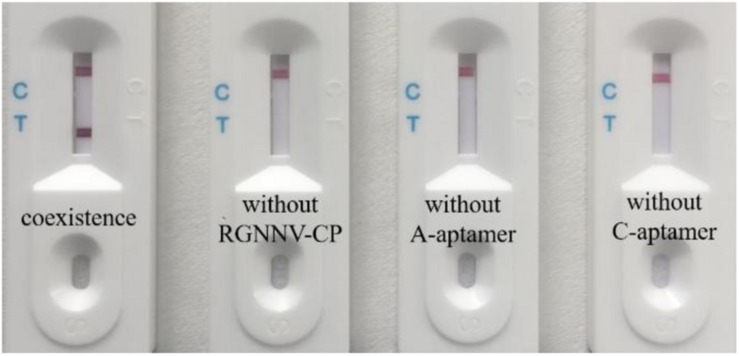
Efficient work of the biosensor for RGNNV-CP detection. From left to right are detection of RGNNV-CP with both A-aptamer and C-aptamer, PBS with both A-aptamer and C-aptamer (control 1), RGNNV-CP with only A-aptamer (control 2) and RGNNV-CP with only C-aptamer (control 3). No positive band was observed in the absence of RGNNV-CP, A-aptamer or C-aptamer. Test line could only be observed obviously in the presence of RGNNV-CP (2 μg/mL) and two aptamers. The experiment was performed in triplicates.

Following assembly of the LFB, we designed the primary experiment (RGNNV-CP with both A-aptamer and C-aptamer) to validate the biosensor for RGNNV-CP detection. Three control experiments were also performed as follows: Control 1, PBS with both A-aptamer and C-aptamer; Control 2, RGNNV-CP (2 μg/mL) with only A-aptamer; Control 3, RGNNV-CP (2 μg/mL) with only C-aptamer. Following SDA, the amplification products were detected with the LFB. Figure 3 shows the detection results.

As the A-aptamer-CP-C-aptamer-SA coated magnetic beads complex could be selected to conduct the next step in the SDA experiment only in the presence of the RGNNV-CP, the SDA ssDNA products were captured by AuNPs–DNA conjugates through the complementary reaction between the AuNPs-DNA (probe 1) and SDA ssDNA products. As shown in Figure 1B, the products were also complementary to probe 3 in the T line; therefore, the products–DNA–AuNPs complex was accumulated in the test zone and visualized as a red band. The excess AuNPs–DNA conjugates continued to migrate, which were captured at the control line as probe 1 on AuNPs was complementary to DNA (probe 2) in the C line, thus forming another red band. In the absence of RGNNV-CP or one of the two aptamers, the A-aptamer was not enriched by magnetic beads, so no SDA product was captured by the T line. Therefore, the positive test line could only be seen in the presence of RGNNV-CP with both A-aptamer and C-aptamer, and no positive band was observed in the absence of RGNNV-CP, A-aptamer or C-aptamer. These results suggested that this biosensor was efficient in the detection of RGNNV-CP.

### Specificity of the Biosensor for RGNNV Detection

**FIGURE 4:**
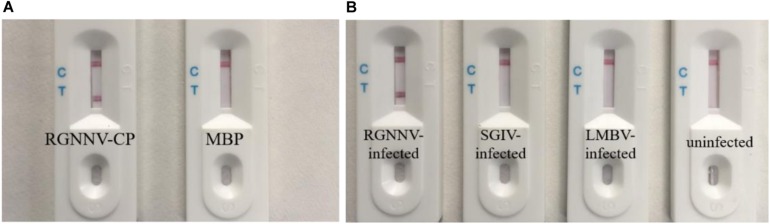
Specificity of the biosensor. **(A)** Results of the biosensor for the detection of RGNNV-CP and control MBP. RGNNV-CP sample can display the visible red line, while MBP did not show a test line. **(B)** Typical images of biosensor for the lysates detection of RGNNV-infected GB cells, SGIV-infected GS cells, LMBV-infected FHM cells and uninfected cells. Only the lysates of RGNNV-infected GB cells were observed with a distinctive test line. The experiment was performed in triplicates.

The specificity of the biosensor was firstly tested using RGNNV-CP and control MBP. As shown in Figure 4A, the RGNNV-CP sample displayed a visible test line, while MBP did not result in a red test line. Virus-infected cell samples were then used to confirm the selection of the biosensor. As shown in Figure 4B, only the lysates of RGNNV-infected GB cells were detected with a distinctive test line. The test zones observed for the lysates of SGIV-infected GS cells, LMBV-infected FHM cells and uninfected cells were similar with a blank test line, which indicated that the biosensor was specific to RGNNV. These results demonstrated that the biosensor had high specificity.

### Optimization of Biosensor Detection Conditions

**FIGURE 5:**
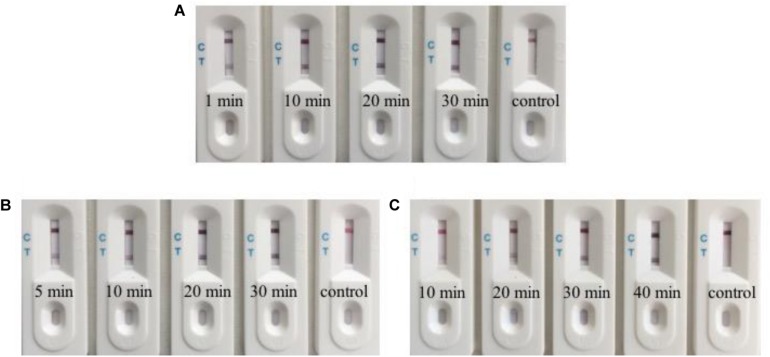
Optimization of the biosensor for detection. **(A)** The biosensor detected RGNNV infection with an incubation time for lysates and aptamers as short as 1 min. **(B)** The binding time of 5 min for magnetic beads and C-aptamer is needed for the biosensor to detect RGNNV infection. **(C)** Typical images of biosensor for RGNNV detection with various SDA reaction time. From 10 to 30 min, the level of redness increased gradually. 30 min is enough to get the optimum visible test line. The experiment was performed in triplicates.

In order to achieve the minimum detection time, we optimized three rate-limiting steps. We collected and lysed 1 × 10^5^ RGNNV-infected GB cells, which were used for biosensor detection. Uninfected GB cells were used as the control. Firstly, we reduced the incubation time of the lysates and two aptamers to determine the effect of incubation time. When the incubation time was decreased to 1 min, the biosensor still showed a red line (Figure 5A). Subsequently, we tested the binding rate of magnetic beads and the C-aptamer (Figure 5B) and it was demonstrated that it took only 5 min for an obvious red line to appear in the test zone. Accordingly, an aptamer incubation time of 1 min and a binding time of 5 min were used in the following experiments. Finally, we examined the SDA products after the following reaction times: 10, 20, 30, and 40 min. The results of different amplification times are shown in Figure 5C. It was found that the level of redness in the test zone increased gradually from 10 min to 30 min, and remained the same color at 40 min. This may have been because the SDA reaction had reached its limit or the amplification materials had run out after 30 min. To obtain the optimum visible test line, SDA for 30 min was chosen for the experiments. Thus, after optimization of the detection procedure, the results were obtained within 1 h.

### Sensitivity of the Assay

**FIGURE 6:**
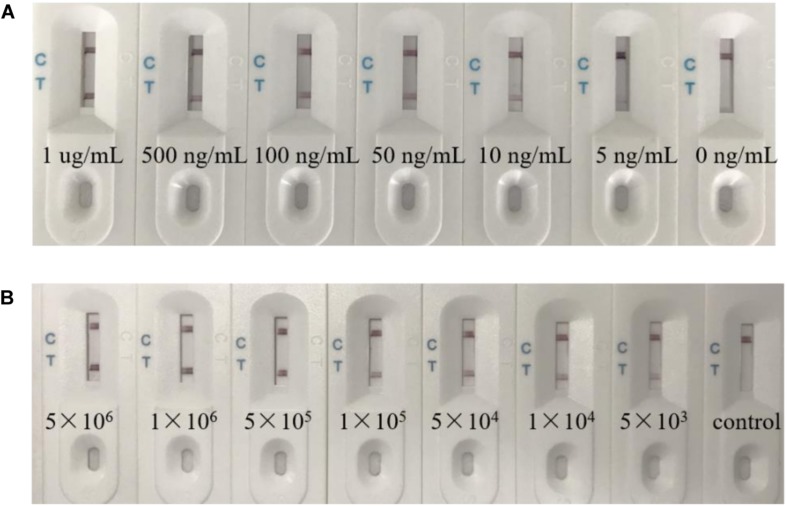
The biosensor had a high sensitivity. **(A)** Typical images of biosensor for RGNNV-CP detection. Seven different concentration samples were detected respectively. The visible line at the test zone could be observed with different concentrations of RGNNV-CP, with the limit being 5 ng/mL. **(B)** Various concentrations of RGNNV-infected GB cells (5 × 10^6^/mL, 1 × 10^6^/mL, 5 × 10^5^/mL, 1 × 10^5^/mL, 5 × 10^4^/mL, 1 × 10^4^/mL, and 5 × 10^3^/mL) were prepared, and uninfected GB cells as the control. The biosensor could detect the lysates of as low as 5 × 10^3^ RGNNV-infected GB cells. The experiment was performed in triplicates.

Firstly, the sensitivity of the biosensor was determined using RGNNV-CP dissolved in PBS at the following concentrations: 1 μg/mL, 500 ng/mL, 100 ng/mL, 50 ng/mL, 10 ng/mL, 5 ng/mL, and 0 ng/mL. Figure 6A shows the results of the biosensor loaded with various quantities of RGNNV-CP. The visible line at the test zone was observed with different concentrations of RGNNV-CP, with the limit being 5 ng/mL. The negative control (0 ng/mL of RGNNV-CP) showed no red line.

In addition, we prepared RGNNV-infected GB cells with various concentrations (5 × 10^6^/mL, 1 × 10^6^/mL, 5 × 10^5^/mL, 1 × 10^5^/mL, 5 × 10^4^/mL, 1 × 10^4^/mL, and 5 × 10^3^/mL) as samples, and uninfected GB cells as the control. As shown in Figure 6B, no visible line was observed in the presence of uninfected GB cells, and the biosensor could detect the lysates of RGNNV-infected GB cells as low as 5 × 10^3^ RGNNV-infected GB cells, which was treated as the limit of detection.

### Real Sample Detection

Twenty groupers with suspected RGNNV infection were tested by RT-PCR and LFB, simultaneously. 12 of these groupers were confirmed to have positive RGNNV infection by RT-PCR. Among them, 10 were confirmed positive by the LFB. In addition, negative samples confirmed by RT-PCR were also negative using the LFB. In general, the results of LFB were a good match with those of RT-PCR, with the sensitivity slightly lower than that of RT-PCR.

## Discussion

Nervous necrosis virus greatly affects aquaculture worldwide around longer historically. Unfortunately, there is no effective strategies to combat NNV infection. Early diagnosis is urgently needed, and attract considerable attention. Toward that goal, LFB combined with aptamer for the rapid detection of RGNNV was set up in the present paper. In this detection system, two aptamers, C-aptamer and A-aptamer, were used to enrich virus and conduct SDA reaction respectively after loading SDA products on the antibody-independent LFB, the detection result was obviously visualized by naked eyes within 5 min. Compared to the common PCR- or antibody-based detection technology, LFB combined with aptamer do not need RNA extraction and cDNA synthesis, do not need the thermal denature and the variable temperature, do not need antibody preparation, do not need the sophisticated manipulation and equipment, and only need shorter time for detection. Hence, it is simple, quick and low cost.

The LFB combined with aptamer displayed good specificity, since T line was visible only when RGNNV-CP or RGNNV was present. The sensitivity of this assay was also performed. The visible T line could be observed by naked eyes with the limitation of 5 ng/mL RGNNV-CP or 5 × 10^3^ RGNNV-infected GB cells. It showed the similar sensitivity as previously reported ELASA methods for RGNNV measurement (4 × 10^3^ RGNNV-infected GB cells lysates), but it was much more convenient and time-saving (Zhou et al., 2017). A previous study showed that the detection limitation of PCR-based LFB was 270 pg of total RNA of NNV. However, it needed almost 4 h to obtain PCR products for follow-up analysis (Toubanaki et al., 2015). While, the detection we used could be completed within 1 h. The lowest limit of detection of one cross-priming isothermal amplification (CPA) based LFB method for RGNNV was 10^1^ copies/μL of RNA solution, which was 10 times lower than that of conventional RT-PCR and comparable to that of RT-qPCR (Su et al., 2015). These two methods still needed RNA extraction and reverse transcription. Another antibodies-based LFB for RGNNV detection showed the detection limit was 10^5.05^ TCID_50_/100 μL without cross-reactivity to other fish viruses (Shyam et al., 2020). By specific scanner, the LFB possibly become more sensitive and provide quantitative data. Hepatitis C virus (HCV) core antigen could be detected in 10 min by aptamer-based LFB, and its lower detection limit was 10 pg/mL with the scanner and 100 pg/mL with naked eyes (Wang et al., 2013).

Apart from RGNNV, many LFB for virus detection have emerged, such as infectious spleen and kidney necrosis virus, cyprinid herpes virus-3, Taura syndrome virus, and white spot syndrome virus (Kiatpathomchai et al., 2008; Jaroenram et al., 2009; Ding et al., 2010; Soliman and El-Matbouli, 2010). LFB is a good choice for pathogen detection outside the laboratory. However, these LFB mostly depends on antibody or PCR products.

In summary, we firstly developed a LFB combined with aptamer-based isolation for the rapid detection of RGNNV. The biosensor showed high detection specificity and sensitivity, independent of RNA extraction and purification steps. The entire detection process can be done in 1 h. Therefore, this biosensor appeared great ability for detecting RGNNV infection. What is more, this method can also be applied in detecting other pathogens in aquaculture, and exhibit great potential to apply in field operation.

## Data Availability Statement

The raw data supporting the conclusions of this article will be made available by the authors, without undue reservation, to any qualified researcher.

## Ethics Statement

All relevant ethical safeguards have been met in relation to animal experimentation, and all animal-involving experiments of this study were approved by the Animal Care and Use Committee of College of Marine Sciences, South China Agricultural University.

## Author Contributions

SW and LZ designed the study. JL performed the experiments, analyzed data, and wrote the main manuscript. QQ helped to design the experiments and supervised all research stages. XZ supplemented some experimental data and made significant advices for the revised version. CL, YY, XH, and OM contributed to the experimental suggestions. All authors read and approved the final manuscript.

## Conflict of Interest

The authors declare that the research was conducted in the absence of any commercial or financial relationships that could be construed as a potential conflict of interest.
