# Supplementary material for: Development of a Novel Lateral Flow Biosensor Combined With Aptamer-Based Isolation: Application for Rapid Detection of Grouper Nervous Necrosis Virus
Source: Front Microbiol. 2020 May 19;11:886. doi: 10.3389/fmicb.2020.00886 (PMC7249735; doi:10.3389/fmicb.2020.00886)
Supplement: Supplementary file 1 [file Data_Sheet_1.pdf]

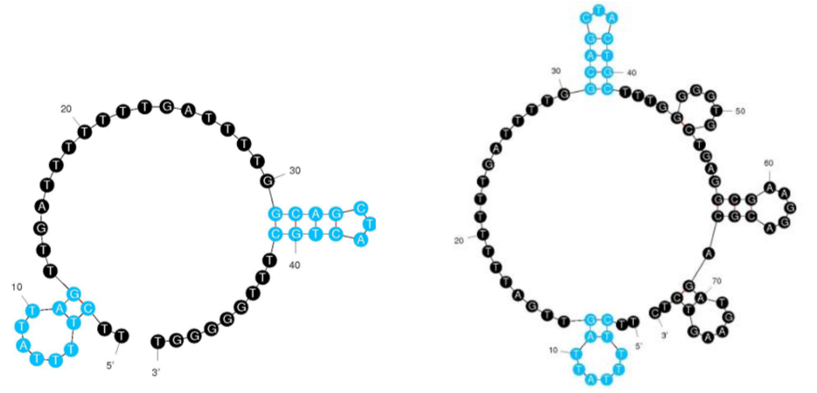

Supplementary Fig. 1. Secondary structures of aptamer B11 and modified-B11 (A-aptamer). Compared with the B11 original sequence (left), the core stem-loop secondary structures still remained in the modified-B11 (right).

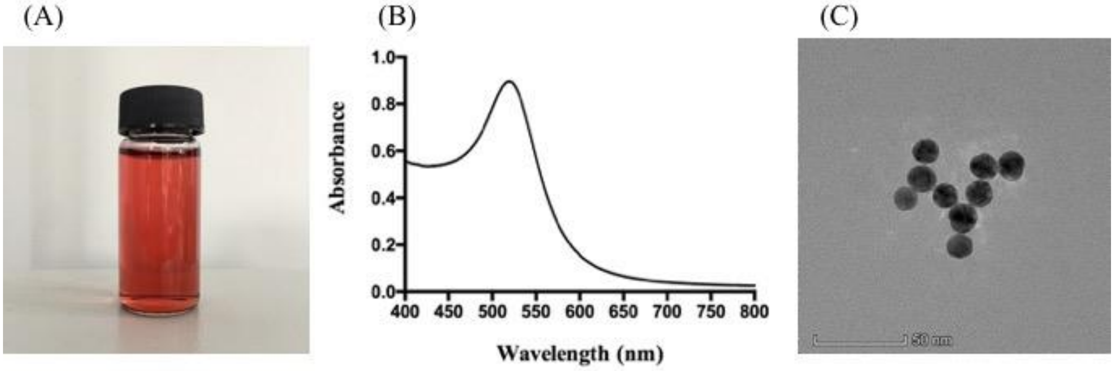

Supplementary Fig. 2. The quality of synthesized AuNPs. (A) The picture of transparent bright red AuNPs solution. There is no suspension at the top and no precipitation at the bottom of the solution. (B) The UV absorbance spectra of AuNPs with the absorption peak at 520 nm. (C) The TEM images of AuNPs. The synthesized AuNPs were spherical in shape and about 15 nm (15.3 ± 0.7 nm) in diameter.
